# Predictors of late gadolinium enhancement cardiac MRI image quality in patients with cardiac implantable electronic devices

**DOI:** 10.1016/j.hroo.2025.07.017

**Published:** 2025-07-29

**Authors:** Farah Amrani, Luuk H.G.A. Hopman, Pieter G. Postema, Michiel J.B. Kemme, Cornelis P. Allaart, Jasper L. Selder, Ramon B. van Loon, Vokko P. van Halm, Marco J.W. Götte, Pranav Bhagirath

**Affiliations:** 1 Department of Cardiology, Amsterdam University Medical Center, Amsterdam, The Netherlands; 2 School of Biomedical Engineering and Imaging Sciences, King’s College London, London, United Kingdom

**Keywords:** Cardiac implantable electronic devices, Late gadolinium enhancement, Image quality, Cardiac magnetic resonance imaging, ICD and pacemaker artifacts, MRI safety

## Abstract

**Background:**

Cardiac implantable electronic devices (CIEDs) can cause artifacts in late gadolinium enhancement (LGE) cardiac magnetic resonance imaging (CMR), compromising diagnostic accuracy. No consensus exists on optimal CIED patient selection for LGE-CMR.

**Objective:**

This study aims to identify predictors of LGE image quality in patients with CIEDS to optimize pre-scan selection.

**Methods:**

Patients with CIEDs who underwent conventional 2D-LGE imaging were retrospectively identified from the Amsterdam UMC CMR database. Baseline clinical and device characteristics were collected, and generator-to-lead distance was measured on post-implantation chest X-rays. LGE quality was categorized as fully diagnostic, acceptable, or non-diagnostic. Multivariable regression and receiver operating characteristic (ROC) analysis determined independent predictors and exploratory generator-to-lead distance thresholds using a 90% sensitivity criterion.

**Results:**

Overall, 80 patients (71.3% male, mean age 64 years) were included: 41.3% ICDs, 23.8% pacemakers (PMs), 23.8% cardiac resynchronization therapy defibrillators (CRT-Ds), and 2.5% cardiac resynchronization therapy pacemakers (CRT-Ps). LGE image quality was fully diagnostic in 48.8%, acceptable in 27.5%, and non-diagnostic in 23.8% of patients. PM/CRT-P patients had no non-diagnostic scans (92.9% fully diagnostic). Only 25.0% of ICD/CRT-D scans were fully diagnostic, while 36.5% were non-diagnostic (*P* < .001). Generator-to-lead distance was significantly associated with LGE quality, with thresholds of 10 cm in ICDs and 8 cm in PMs for acceptable LGE quality.

**Conclusion:**

Device type and positioning significantly impact LGE image quality. ICDs were associated with poorer image quality, while PMs consistently yielded diagnostic-quality images. Generator-to-lead distance emerged as a key predictor, providing a practical tool for optimizing LGE-CMR referrals. This study defines generator-to-lead distance thresholds by device type and proposes a structured pre-scan workflow to support LGE-CMR referral decisions in patients with CIEDs.

## Graphical abstract

**Figure:**
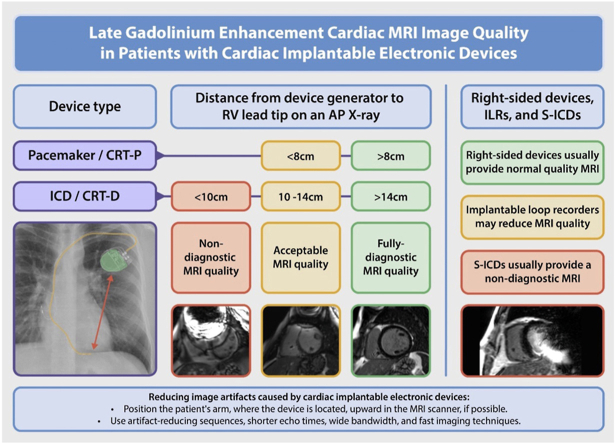

**Key Findings**

▪ Generator-to-lead distance is a practical and reproducible metric for estimating late gadolinium enhancement (LGE) cardiac magnetic resonance imaging (CMR) quality in patients with cardiac implantable electronic devices (ICDs).
▪ Device type strongly influences artifact burden, with ICDs associated with significantly lower diagnostic yield compared to pacemakers.
▪ A generator-to-lead distance of ≥97 mm for ICD/cardiac resynchronization therapy (CRT)- defibrillator and ≥78 mm for pacemaker/CRT-pacemaker predicted acceptable or diagnostic-quality scans with 90% sensitivity.
▪ A structured imaging referral workflow that integrates post-implant chest radiography, device type, and clinical context to guide LGE-CMR use can potentially reduce non-diagnostic studies and improve resource use.

## Introduction

Cardiac magnetic resonance (CMR) imaging is increasingly performed in patients with cardiac implantable electronic devices (CIEDs), including pacemakers (PMs) and implantable cardioverter defibrillators (ICDs).^1^ Advances in technology, such as the widespread adoption of MR-conditional devices and evidence supporting the safety of CMR in non-MR-conditional devices and abandoned leads, have significantly expanded eligibility for this imaging modality.^2^, ^3^, ^4^, ^5^ CMR plays a pivotal role in the management of patients with CIEDs, as it provides high spatial and temporal resolution images, enabling detailed assessment of tissue characteristics, functional parameters, structural abnormalities, and inflammatory processes.^6^ This facilitates the diagnosis of cardiac pathologies, guiding decisions about device upgrades or extractions, and mapping arrhythmogenic substrates for ventricular tachycardia ablation.^7^^,^^8^

Among CMR techniques, late gadolinium enhancement (LGE) imaging has become a cornerstone for detecting myocardial fibrosis and scar, providing crucial insights for disease characterization and procedural planning.^9^^,^^10^ However, in patients with CIEDs, achieving high-quality LGE imaging is often challenging due to device-related artifacts.^11^^,^^12^ These artifacts result from electromagnetic interference and magnetic susceptibility differences, leading to signal voids and image distortion, particularly in regions near the device generator.^13^ Such artifacts degrade image quality, limiting diagnostic utility and raising concerns about the effort and risks of CMR in these patients. Challenges include the need for specialized staff, such as device technicians, prolonged imaging times, and temporary deactivation of ICD functions during scanning. Despite advancements that have improved the safety of CMR in patients with MR-conditional devices, these challenges persist.

Although prior studies, such as Kocyigit et al,^14^ have described associations between generator-to-lead distance and CMR artifacts, these findings remained largely observational, lacked stratification by device type, and did not offer clinically actionable thresholds.

This study aims to address this gap by identifying predictors of LGE image quality in patients with CIEDs with specific cutoff values to optimize patient selection. By integrating these findings into a referral workflow, we expect to provide a novel and practical decision support tool for optimizing scan selection and resource use.

## Methods

### Study population

This retrospective study analyzed patients with CIEDs who underwent CMR imaging, including LGE acquisition at the Amsterdam University Medical Center. The study was conducted in accordance with relevant ethical standards, and institutional permission was granted for access to anonymized clinical data. Informed consent was obtained where applicable. Patients were identified from the institutional imaging database, and those with subcutaneous ICDs (S-ICD), implantable loop recorders (ILRs), right-sided devices, or incomplete data (no post-CIED implant chest X-ray) were excluded. Patient characteristics, including demographics, clinical history, and post-CIED implantation chest X-ray parameters, were retrieved from electronic medical records. Device characteristics, such as manufacturer and implantation date, were also collected. Left ventricular end diastolic diameter (LVEDD) was assessed from a pre-CMR echocardiogram, if available.

### LGE image acquisition

All CMR examinations were conducted using 1.5T MRI scanners (Siemens Aera or Avanto, Healthineers, Erlangen, Germany). LGE images were obtained using segmented, breath-held inversion recovery sequences in the short-axis orientation, typically 10–15 minutes after administration of 0.2 mmol/kg gadolinium-based contrast agent (Dotarem, Guerbet, Villepinte, France). Inversion time (TI) was selected using a Look-Locker scout. No wideband LGE or 3D LGE sequences were used in this retrospective analysis. Similarly, patient arm positioning during CMR (arms elevated vs by the side) was not standardized or documented. Imaging parameters included spatial resolution of 1.3 × 1.3 × 8 mm, repetition time 782 ms, echo time 4.38 ms, and flip angle 25°.

### X-ray analysis

Post-CIED implantation chest X-rays were analyzed in a posteroanterior projection to measure the distance between the device generator and the right ventricular (RV) lead tip. Post-implantation chest X-rays were routinely acquired in the upright position during expiration, in accordance with institutional protocol. Since CMR is performed supine, we acknowledge the potential anatomical variation, but chose the most routinely available view for clinical applicability.

### Image quality assessment

LGE image quality was evaluated by 2 expert readers blinded to clinical data (LH and PB). Reviewers had 5 and 10 years of experience in cardiac MRI, respectively. Images were assessed on a 17-segment myocardial model and categorized into 3 quality groups:

- Fully diagnostic: No artifacts; all segments interpretable.
- Acceptable: Minimal artifacts present but diagnostic utility maintained; ≥50% of segments interpretable. The ≥50% cutoff was chosen to define “acceptable” image quality, in line with prior studies using segment-based interpretability thresholds.
- Non-diagnostic: Artifacts obscuring >50% of the myocardium, rendering images unsuitable for diagnostic interpretation.

Segmental analyses were performed to quantify the number of interpretable segments per study. Final image quality classification was determined by consensus.

### Statistical analysis

Continuous variables were expressed as mean ± standard deviation or median (range), depending on distribution assessed by the Kolmogorov-Smirnov test. Categorical data were presented as frequencies and percentages. The relationship between image quality categories (fully diagnostic, acceptable, non-diagnostic) and predictor variables was evaluated using Spearman’s correlation and χ^2^ tests.

Differences between predictors across image quality categories were assessed using one-way analysis of variance with Bonferroni post-hoc corrections for multiple comparisons. Multivariable regression analysis was performed to identify independent predictors of image quality, adjusting for potential confounders. Receiver operating characteristic (ROC) curves were constructed to evaluate the area under the curve (AUC) for the prediction of acceptable or fully diagnostic LGE image quality, using generator-to-lead distance as the primary predictor. Given the retrospective design and limited sample size, all ROC analyses were exploratory and not validated in a separate cohort. A sensitivity threshold of 90% was selected based on clinical considerations, prioritizing the need to identify nearly all patients likely to achieve acceptable or fully diagnostic image quality while minimizing the risk of false exclusions. This approach ensures that most patients who could benefit from LGE-CMR are considered for referral, even if it results in a higher number of scans, aligning with the study’s goal of optimizing patient selection for LGE-CMR.^15^

All analyses were performed using IBM SPSS Statistics (version 29.0; IBM Corp, Armonk, NY). A two-tailed *P*-value <0.05 was considered statistically significant.

## Results

**Figure 1:**
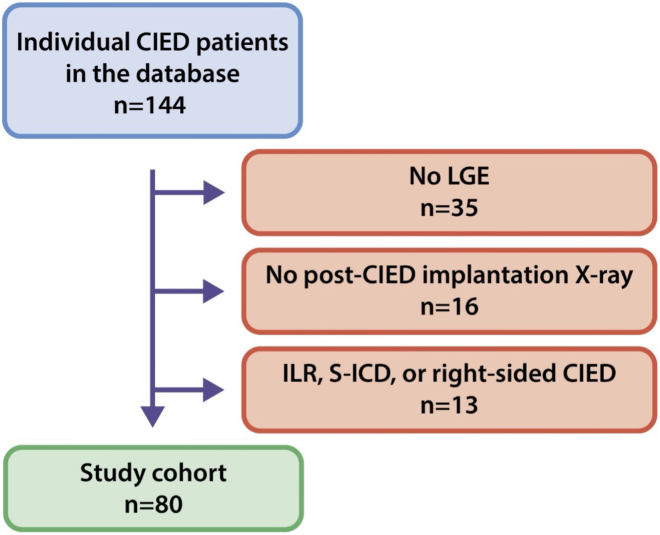
Patient selection flowchart for CIED undergoing LGE-CMR. Flowchart depicting the patient selection process. Exclusion factors included absence of LGE imaging, absence of post-implant X-ray, or type of device. CIED = cardiac implantable electronic device; LGE = late gadolinium enhancement; ILR = implantable loop recorder; S-ICD = subcutaneous ICD.

A total of 80 patients were included in the study (Figure 1), with baseline characteristics summarized in Table 1. The mean age was 63 years, and 71.2% were male. Of these, 33 patients (41.3%) had ICDs, 26 (32.5%) had cardiac resynchronization therapy defibrillator (CRT-D) devices, 19 (23.8%) had pacemakers (PM), and 2 (2.5%) had cardiac resynchronization therapy pacemaker (CRT-P) devices. Myocardial scar was observed in approximately 79% of ICD/CRT-D patients and in 64% of those with PM/CRT-P devices.

**Table 1:** Baseline characteristics

Baseline characteristics	
Age (years)	63 ± 13
Gender (male, n [%])	57 (71.2%)
Body mass index	27.4 ± 5.4
LVEDD on echo	55.9 ± 10.1
Distance device to RV lead tip (mm)	122.7 ± 35.1
Etiology	
- Ischemic cardiomyopathy	22 (27.5%)
- Non-ischemic cardiomyopathy	58 (72.5%)
Device type n (%)	
ICD	33 (41.3%)
PM	19 (23.8%)
CRT-D	19 (23.8%)
CRT-P	2 (2.5%)

CRT-D indicates cardiac resynchronization therapy defibrillators; CRT-P = cardiac resynchronization therapy pacemakers; ICD = implantable cardioverter defibrillators; LVEDD = left ventricular end diastolic diameter; PM = pacemakers; RV = right ventricular.

### LGE image quality

Of the 80 CMR scans evaluated, 39 (48.8%) were classified as fully diagnostic, 22 (27.5%) as acceptable, and 19 (23.8%) as non-diagnostic. On average, acceptable-quality scans contained 5.0 ± 1.0 non-diagnostic myocardial segments, whereas non-diagnostic scans had 11.4 ± 3.0 non-diagnostic segments.

When stratified by device type, significant differences in image quality were observed. Fully diagnostic scans were more frequently achieved in patients with PM compared to patients with ICD (92.9% vs 25.0%, *P* < .001). None of the patients in this cohort had left bundle branch pacing (LBBP) systems. Conversely, non-diagnostic scans were significantly more common in ICD/CRT-D patients (36.5%), whereas no non-diagnostic scans were observed in the PM/CRT-P group (*P* < .001).

### Predictors of image quality

Multivariable regression analysis identified the distance from the device generator to the RV lead tip as the sole significant predictor of LGE image quality in the total cohort (*P* = .001). Fully diagnostic scans had a significantly greater mean generator-to-lead distance (135.5 ± 35.7 mm) compared to acceptable (110.4 ± 28.4 mm) and non-diagnostic scans (110.8 ± 33.0 mm). In contrast, body mass index (BMI) and LVEDD were not significantly associated with image quality. The mean BMI was 27.3 ± 5.3 kg/m^2^ in fully diagnostic scans, compared to 28.6 ± 6.4 kg/m^2^ in acceptable and 26.2 ± 4.1 kg/m^2^ in non-diagnostic scans (*P* = .355). Similarly, LVEDD values were comparable across groups (54.2 ± 9.3 mm for fully diagnostic, 59.9 ± 8.8 mm for acceptable, and 54.8 ± 12.2 mm for non-diagnostic scans, *P* = .078).

When stratified by device type, ICD/CRT-D patients exhibited a stronger dependence on generator-to-lead distance (*P* = .087, mean: 116.8 ± 30.4 mm), whereas PM/CRT-P patients did not show a significant association (*P* = .074, mean: 133.7 ± 40.9 mm). Neither BMI (*P* = .059–.355) nor LVEDD (*P* = .078–.080) was a significant predictor in either device group. Gender was not a significant predictor of image quality (χ^2^ = 2.52, *P* = .283).

### ROC analysis

**Figure 2:**
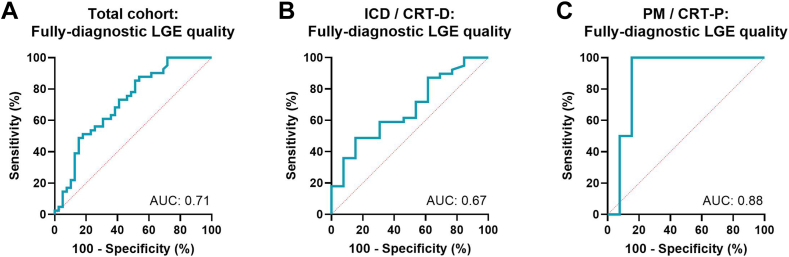
Receiver operating characteristic curves for predicting acceptable or fully diagnostic LGE image quality. Receiver operating characteristic (ROC) curves assessing the predictive performance of generator-to-lead distance for acceptable or fully diagnostic LGE image quality. The area under the curve (AUC) was calculated to quantify the discriminative ability of generator-to-lead distance. Panel A, ROC curve for the total cohort (AUC = 0.714, 95% CI: 0.615–0.813); Panel B, ROC curve for the defibrillator subgroup (AUC = 0.672, 95% CI: 0.544–0.799); Panel C, ROC curve for the pacemaker subgroup (AUC = 0.885, 95% CI: 0.766–1.000).

ROC analysis was performed to evaluate the ability of generator-to-lead distance to predict acceptable or fully diagnostic LGE image quality. In the total cohort (Figure 2A), the ROC analysis yielded an AUC of 0.714 (95% confidence interval [CI]: 0.615–0.813), indicating moderate predictive accuracy. The optimal generator-to-lead distance for predicting acceptable or fully diagnostic scans with 90% sensitivity was 80 mm.

When stratified by device type, the ICD/CRT-D group demonstrated an AUC of 0.672 (95% CI: 0.544–0.799), with an optimal cutoff distance of 97.4 mm for 90% sensitivity in predicting fully diagnostic scans (Figure 2B). In contrast, the PM/CRT-P group exhibited stronger predictive performance, with an AUC of 0.885 (95% CI: 0.766–1.000) (Figure 2C). The optimal generator-to-lead distance for this group was 77.9 mm for 90% sensitivity.

## Discussion

This study evaluated the impact of CIEDs on LGE-CMR image quality and identified the distance between the device generator and the RV lead tip on post-implantation chest X-rays as the sole significant predictor. This distance offers a practical, clinically applicable metric for guiding patient selection and managing expectations regarding LGE-CMR quality. To our knowledge, this is the first study to define specific, ROC-derived cutoff values stratified by device type for predicting diagnostic image quality using chest radiography. Moreover, by incorporating these thresholds into a structured pre-scan referral workflow, this work advances beyond earlier descriptive studies and provides a pragmatic tool to inform clinical decision-making.

### Impact of device type on image quality

Our findings underscore the significant influence of CIED type on LGE image quality. PMs were consistently associated with acceptable or fully diagnostic LGE images, with only 2 cases classified as acceptable and none as non-diagnostic. In contrast, ICDs exhibited poorer image quality due to more pronounced artifacts. Artifacts from the generator predominantly affected the anterolateral myocardial wall, aligning with its physical position and electromagnetic impact. Although no patients in our cohort had LBBP, the typically higher septal lead position associated with LBBP may reduce the generator-to-lead tip distance and could theoretically influence LGE image quality. These artifacts result from magnetic susceptibility differences and signal voids caused by the metallic casing and internal components of the device, with larger artifacts observed in ICDs due to their thicker, more complex design compared to PMs.^16^

Although our study primarily focused on device type and generator-to-lead distance as predictors of image quality, we acknowledge that scar location may also play an important role in determining diagnostic interpretability. Artifacts from CIEDs tend to cluster in the anterolateral myocardial wall, and it is conceivable that scar located in these artifact-prone regions may be obscured or mischaracterized. Although scar location was systematically documented, the sample size and scar distribution did not allow for a reliable subgroup analysis comparing LGE image quality across different scar localizations (eg, septal vs lateral wall). Nonetheless, this interplay between scar distribution and artifact localization remains clinically relevant, particularly in patients referred for substrate-based interventions, such as ventricular tachycardia (VT) ablation, where precise scar visualization is critical. We have identified this as an important area for future investigation, ideally in larger, prospectively designed cohorts.

Beyond regional scar distribution, the overall prevalence of myocardial scar also differed by device group, with a higher proportion observed in ICD/CRT-D recipients compared to those with PM/CRT-P systems. Although not directly assessed in this study, such differences in underlying myocardial pathology may influence diagnostic yield, especially when diffuse or extensive scar coincides with artifact-prone areas.

### Predictive value of X-ray measurements

The generator-to-RV lead tip distance emerged as the sole significant predictor of diagnostic image quality. Exploratory ROC analysis suggested threshold distances of approximately 97.4 mm (10 cm) for ICD/CRT-D devices and 77.9 mm (8 cm) for PM/CRT-P devices at 90% sensitivity. These findings underscore the value of this simple, noninvasive metric in pre-scan patient assessment. However, the moderate AUC (0.672 for ICD/CRT-D) suggests that additional factors influence image quality, including device manufacturer, the insulating effect of surrounding tissues such as fat, and variations in patient anatomy.^17^ Furthermore, reliance on post-implantation chest radiographs introduces limitations, as device positioning may shift over time due to migration or changes in body composition. Obtaining a chest radiograph as part of the pre-CMR evaluation in patients with CIEDs may thus be useful in assessing the current generator-to-heart distance. Similar to an orbital radiograph in patients at risk for intraocular metal, this straightforward pre-scan assessment may aid in managing expectations for LGE image quality.

In addition to generator-to-lead distance, lead location within the right ventricle may also influence artifact severity, particularly when the lead is positioned along a more septal trajectory and closer to the generator. While most leads in our cohort were placed in the apex or distal septum, further study is warranted to assess whether variations in lead orientation contribute meaningfully to LGE image quality.

**Figure 3:**
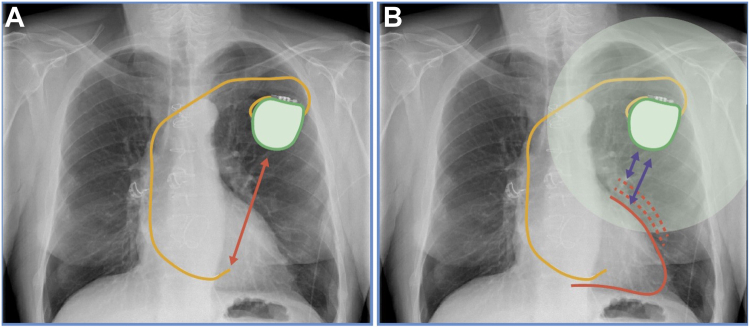
Potential impact of left ventricular dilation on LGE-CMR image quality in CIED patients. Illustration of the potential relationship between left ventricular (LV) dilation and LGE-CMR image quality in patients with CIEDs. One proposed factor influencing the predictive value of generator-to-RV lead tip distance is LV dilation, which may alter myocardial positioning relative to the device generator. Panel A illustrates a normal-sized left ventricle, with greater myocardial-to-generator distance, potentially reducing artifact impact. Panel B, *dotted red lines* show an increasingly dilating left ventricle, where anterior wall displacement towards the generator region may result in more severe artifacts, potentially compromising LGE-CMR interpretability.

Another factor that may have influenced the predictive value of generator-to-RV lead tip distance is LV dilation. Although this was not supported by our multivariable analysis, it is plausible that patients with dilated cardiomyopathy experience progressive LV enlargement, bringing the anterior myocardial wall closer to the generator’s artifact-prone region (Figure 3). Pre-scan echocardiography could provide valuable insights into the impact of LV dilation on image quality.

Despite these nuances, the findings offer actionable insights for optimizing LGE-CMR imaging in patients with CIEDs. Importantly, however, although the generator-to-RV lead tip distance serves as a straightforward predictor of image quality, clinical indications and patient-specific characteristics must ultimately guide decisions. For instance, even in cases where anterior myocardial wall artifacts are expected, LGE-CMR may still provide critical diagnostic information, such as identifying inferior infarctions or arrhythmogenic substrates.

### Comparison with prior literature

Previous studies, for example, Kocyigit et al,^14^ have described the general relationship between CIED positioning and MRI artifact, introducing a Device-Related Score to estimate imaging limitations. However, these studies did not provide exploratory distance thresholds based on diagnostic interpretability, nor did they stratify results by device subtype (ICD vs PM). Our study builds on this work by offering concrete, stratified cutoff values derived from ROC analysis and embedding these into a decision-making framework. We also extend these findings by quantifying image quality on a segmental level and analyzing diagnostic yield, thereby enhancing their clinical relevance.

### Advances in mitigating device-related artifacts

While artifacts from CIEDs remain a challenge, advances in imaging techniques have significantly reduced their impact. For example, positioning the patient’s arm above their head during scanning increases the distance between the generator and the myocardium, improving image quality. Furthermore, the increasing availability of MRI-conditional devices has also expanded the pool of patients eligible for safe and effective imaging. Additionally, experienced centers can safely image non-conditional devices by following established safety protocols.^18^

Wideband LGE imaging is a particularly promising development, as it addresses susceptibility artifacts by correcting for device-related frequency shifts. This technique has shown significant improvements in image quality in artifact-prone regions, though its current availability is largely restricted to research institutions.^19^ Emerging 3D LGE imaging techniques, possibly combined with broad bandwidth technology, also hold potential for enhanced scar visualization in cases of inferior wall infarctions or arrhythmia substrate characterization.^20^

## Limitations

This study has several limitations. First, the analysis was conducted using a single MRI platform and imaging protocol, which may limit the generalizability of the findings to other scanners or institutions. Future research should aim to standardize imaging protocols across different MRI systems to address this variability.

Second, while the generator-to-lead distance was identified as a significant predictor, the moderate AUC highlights the need to investigate additional contributing factors, such as patient anatomy, device-specific features, or changes in device position over time. Additionally, since no PM scans were classified as non-diagnostic in our dataset (and only 2 as acceptable), we cannot determine whether generator-to-lead distance meaningfully predicts poor image quality in PM recipients. Future studies with larger sample sizes and a wider range of PM imaging outcomes are needed to explore this further.

Our analysis also did not include data on device manufacturer, lead type or size, or device position over time, which may influence artifact severity. Furthermore, patient positioning (eg, arm elevation) and use of alternative imaging techniques (wideband or 3D LGE) were not standardized or captured retrospectively. Lastly, S-ICDs were excluded from this analysis, leaving an important gap in our understanding of their impact on LGE-CMR image quality.^21^ Further studies are warranted to explore these devices and validate the findings across larger, more diverse populations.

## Conclusion

This study underscores the significant influence of device type and positioning on LGE-CMR image quality in patients with CIEDs. The generator-to-RV lead tip distance serves as a practical and clinically applicable predictor of imaging outcomes, with exploratory distance thresholds for PMs and ICDs to achieve optimal diagnostic quality. However, the moderate predictive value of this measure highlights that other factors also play a role in determining image quality. These may include device-specific characteristics, patient anatomy, and the time elapsed since implantation, all of which warrant further investigation. Advances such as wideband LGE and 3D imaging hold promise for further improving image quality and arrhythmia substrate characterization in this growing patient population.

## Disclosures

The authors have no relevant conflicts of interest to disclose.
